# Upregulation of claudin‑4 by Chinese traditional medicine Shenfu attenuates lung tissue damage by acute lung injury aggravated by acute gastrointestinal injury

**DOI:** 10.1080/13880209.2022.2128824

**Published:** 2022-10-13

**Authors:** Yueliang Zheng, Mian Zheng, Jing Shao, Chengxing Jiang, Jian Shen, Rujia Tao, Yuqin Deng, Yingge Xu, Yuanqiang Lu

**Affiliations:** a Department of Emergency Medicine, The First Affiliated Hospital, School of Medicine, Zhejiang University, Hangzhou, People’s Republic of China; b Emergency & Intensive Care Unit Center, Department of Emergency Medicine, Zhejiang Provincial People’s Hospital, Affiliated People’s Hospital, Hangzhou Medical College, Hangzhou, China; c School of Medicine, Zhejiang University, Hangzhou, China; d Cancer Institute of Integrated Traditional Chinese and Western Medicine, Zhejiang Academy of Traditional Chinese Medicine, Tongde Hospital of Zhejiang Province, Hangzhou, China

**Keywords:** Inflammation, intestinal barrier injury, p38/ERK phosphorylation

## Abstract

**Context:**

Many studies have explored new methods to cure acute lung injury (ALI); however, none of those methods could significantly change the high mortality rate of ALI. Shenfu is a Chinese traditional medicine that might be effective against ALI.

**Objective:**

Our study explores the therapeutic potential of Shenfu in ALI.

**Materials and methods:**

Male C57BL/6 mice were assigned to control, lipopolysaccharide (LPS) (500 µg/100 μL per mouse), and LPS + Shenfu (30 mL/kg) groups. Shenfu (10 µL/mL) was added to LPS (10 µg/mL) treated MLE-12 cells for 48 h *in vitro*. Male C57BL/6 mice were divided into four groups: LPS, LPS + 3% dextran sulphate sodium (DSS), 3% DSS + Shenfu, and LPS + 3% DSS + Shenfu.

**Results:**

Compared with the ALI group, Shenfu reduced wet/dry weight ratio (19.8%, 36.2%), and reduced the IL-2 (40.9%, 61.6%), IFN-γ (43.5%, 53.3%) TNF-α (54.1%, 42.1%), IL-6 (54.8%,70%), and IL-1β (39.9%, 65.1%), reduced serum uric acid (18.8%, 48.7%) and creatinine (17.4%, 41.1%). Moreover, Shenfu enhanced cell viability (17.2%, 59.9%) and inhibited cell apoptosis (63.0%) and p38/ERK phosphorylation in *in vitro* cultured epithelial cells with LPS stimulation. Mechanistically, Shenfu mediated the protective effect by upregulating claudin-4 expression. In addition, Shenfu could protect against both lung and intestinal epithelial damage in acute gastrointestinal injury-exacerbated ALI.

**Discussion and conclusions:**

Taken together, the results revealed the therapeutic effect and the underlying mechanism of Shenfu injection in an ALI in mouse model, indicating its clinical potential to treat patients with ALI.

## Introduction

Acute lung injury (ALI) is a common clinical syndrome of an acute systemic inflammatory process in the lung that disrupts endothelial and epithelial barriers (Rubenfeld et al. 2005; Johnson and Matthay 2010; Schmidt and Tuder 2010). ALI and its most severe form, acute respiratory distress syndrome (ARDS), are the major causes of morbidity and mortality in critical patients. The incidence of ALI is about 17–34 annual cases per 100,000 people, with mortality rates of 34–58% (MacCallum and Evans 2005). Common causes of ALI/ARDS are sepsis, trauma, shock, aspiration, inhalation injury, multiple blood transfusion, acute pancreatitis, and even certain drug toxicities. Direct or systemic lung injury might initiate the disease process, while the related inflammatory response propagates lung injury, especially when the disease is coupled with other insults related to the lung. In the early stage of ALI, accumulated tissue damage of the endothelial and epithelial barriers increases lung permeability, which causes alveolar edoema (Matthay et al. 2000). Then, inflammatory responses characterized by secretion of inflammatory cytokines [e.g., tumour necrosis factor alpha (TNF-α), interleukin (IL)-6, and IL-1β] and influx of neutrophils to the alveolar space are activated (Ward 2003). This acute inflammation enhances tissue damage, which in turn aggravates the disease. Meanwhile, fibroproliferation occurs in the alveolar hyaline membranes, as well as varying degrees of interstitial fibrosis.

Based on the different insults, ALI with primary lung damage is classified as the pulmonary type, whereas, ALI, without primary lung injury, is classified as the extrapulmonary type (Pelosi et al. 2003). Extrapulmonary diseases, such as those in the brain, intestine, heart, liver, or kidney, contribute to the development of ALI. Meanwhile, ALI also serves as a risk factor for various diseases in extrapulmonary organs. All the evidence suggests the existence of interorgan networks. This is consistent with the viewpoint of traditional Chinese medicine that there is a close correlation between the lungs and the intestines. Currently, this opinion is supported by many studies of organ crosstalk in the lung-gut axis. Correlation analysis of irritable bowel syndrome (IBS), one of the most common diseases of the gastrointestinal tract, suggested that IBS is associated with bronchial hyper-responsiveness (Yazar et al. 2001). Patients with bronchial asthma also had an increased prevalence of IBS (Roussos et al. 2003). Changes in intestinal mucosal structure and function were observed in patients with asthma. Moreover, patients with chronic obstructive pulmonary disease (COPD) had increased intestinal permeability and were 3-fold more likely to be diagnosed with inflammatory bowel disease (IBD) (Rutten et al. 2014). Studies revealed that the bidirectional lung-gut axis was mediated by multiple mechanisms, including the microbiome. It was observed in sepsis and ARDS, bacteria could transfer from the intestines to the lungs (Dickson et al. 2016). Segmental filamentous bacteria (SFB) in mouse intestines can stimulate pulmonary T-helper (Th)17 responses and protect mice from SFB lung infection (Gauguet et al. 2015). Intestinal epithelial cells and immune cells stimulated by microbes could trigger local cytokine responses and induce inflammatory responses, finally triggering the distal immune responses in the lung (Trompette et al. 2014). These results suggested factors including microbe seeding, metabolites, circulatory immune cell, and cytokines, established the relationship between the gut and the lung.

Accumulated knowledge suggested that resolution of ALI includes not only relief from injurious factors but also promotion of actively regulated programs, including removal of apoptotic neutrophils, remodelling of the tissue matrix, clearance of protein-rich alveolar fluid, and the engagement of numerous signalling pathways related to ALI recovery (Tsushima et al. 2009). In past decades, many pharmacological strategies have been explored in clinical trials. However, few of them proved to be effective to reduce mortality. Currently, the most common type of therapy for patients with ALI is still supportive care, such as mechanical ventilation. Therefore, there is an urgent need to develop more effective drugs with anti-inflammatory and tissue-protective efficacy to treat ALI.

Clinical outcomes of patients with ALI are strongly associated with the severity of the damage to the alveolar barrier. This layer of the alveolar epithelium maintains a surface for gas transfer. It has a central role in keeping maintaining lung function. However, it is easily exposed to potentially hazardous stimuli. Therefore, recent work has focussed on the alveolar epithelium because of its centrality to ALI, as well as several other lung diseases. Following the discovery of tight junctions, the field of barrierology emerged, which drew increased attention to the function and regulation of tight junction proteins of epithelial barriers (Sawada et al. 2003). Claudin family proteins were identified as major structural components of the tight junction. They are integral membrane proteins found in tight junctions of all epithelia and endothelia (Gunzel and Yu 2013). Studies showed that claudins constitute both paracellular barriers and pores. Claudins also play a key role in determining the permeability properties of epithelial and endothelial cells. Human lung tissue expresses claudins 1, 3, 4, 5, 7, 8, 10, 15, and 18. In alveolar cells, claudins 3, 7 and, especially, claudin-4 are the predominantly expressed members (Kaarteenaho-Wiik and Soini 2009). It has been suggested that in the lung, claudin-4 acts either as a general barrier or as an alveolar Na + barrier, preventing the leakage of electrolytes and fluid into the alveolar space (Kaarteenaho-Wiik and Soini 2009). In a mouse ALI model, claudin-4 was observed to be induced specifically among the tight junction proteins, which was dependent on protein kinase C (PKC) activation and the JUN N-terminal kinase (JNK)-mitogen activated protein kinase (MAPK) pathway (Wray et al. 2009). Moreover, removal of claudin-4 from the tight junction exacerbated ventilator-induced pulmonary edoema via inhibition of air space fluid clearance in an ALI mouse model (Wray et al. 2009). Consistent with this, claudin-4 expression showed a strong positive correlation with alveolar fluid clearance in *ex vivo*-perfused human lung specimens (Rokkam et al. 2011). Except in the lung, claudin-4 downregulation was observed under various conditions that caused increased permeability of colon tissue, indicating that claudin-4 also has the same role in the colon as in the lung (Gunzel and Fromm 2012). These results proved that claudin-4 plays a central role in the maintenance of the normal epithelial barrier function as well as in recovery of the epithelium from tissue injury-related diseases, especially of ALI. This suggested that medication that enhances claudin-4 function could have the potential to treat ALI.

Shenfu is a traditional Chinese herbal medicine extracted from the roots of *Panax ginseng* C.A. Mey (Araliaceae) and *Aconitum carmichaelii* Debeaux (Ranunculaceae). It is widely used as an anti-inflammatory reagent to treat endotoxin shock and heart disease in China. The major active ingredients include ginsenoside and aconite total alkaloids (Zhu et al. 2013). Many clinical reports indicated that Shenfu has a rapid effect in emergency use, with functions including strengthening the heart, improving heart function, protecting vascular endothelial cells, regulating immune function, anti-inflammation, and improving hypoxia tolerance (Liu C et al. 2015). Moreover, injection of Shenfu was officially approved in 1987 in China (approval code: Z20043117). An animal study showed that Shenfu injection suppressed lung tissue damage in an endotoxin shock-induced rat ALI model. Administration of Shenfu improved the survival rate of rats with endotoxin shock-induced ALI, possibly through suppression of the high mobility group box 1 (HMGB1)-nuclear factor kappa B (NF-κB) pathway and inhibition of secretion of proinflammatory cytokines such as TNF-α and IL-1β (Liu X et al. 2019). In another study, damaged ileal mucosal villi, discontinuous tight junctions between epithelial cells, and broken organelles and microvilli were detected in a caecal ligation and puncture rat sepsis model. In that model, Shenfu injection increased the expression of tight junction markers, including claudin 3 and zona occluddens-1 (ZO-1), and significantly alleviated intestinal epithelial damage. An overt improvement was observed in the treatment group: tight junctions were clearly visible between intestinal epithelial cells (Xing et al. 2015). Using the same sepsis model, another group demonstrated that Shenfu injection attenuated peritoneal inflammation, relieved sepsis-induced intestinal permeability, and prevented intestine and liver damage via regulation of ZO-1, occludin, claudin 1, and vasodilator-stimulated phosphoprotein (Liu et al. 2021). These results explained the tissue protective aspect of Shenfu, at least partially, as regulation of tight junctions of the epithelial barrier and anti-inflammation.

Based on the therapeutic aspects of Shenfu, we proposed that Shenfu injection might be a potential treatment for ALI. We hypothesized that Shenfu injection could upregulate tight junction proteins such as claudin 4, thus rescuing damage to the alveolar barrier, which in turn would control lung inflammation and help the relief and recovery of mice from ALI. In the present study, our research supported this hypothesis by: (1) demonstrating that Shenfu injection indeed alleviates inflammatory responses and tissue damage in lipopolysaccharide (LPS)-induced ALI; (2) showing *in vivo* that Shenfu injection has a key role in the regulation of tight junction proteins and in the maintenance of epithelial barriers; (3) showing *in vitro* that the protective role of Shenfu in ALI is dependent on the regulation of claudin 4; and (4) Shenfu exerts a systemic protective function towards the lung and intestines in acute gastrointestinal injury (AGI)-exacerbated ALI. This study provides new targets and an up-front experimental basis for the treatment of ALI.

## Materials and methods

### Animals

All mice were purchased from Ziyuan Co. (Hangzhou, China) and maintained in the animal facility of the First Affiliated Hospital, School of Medicine, Zhejiang University under pathogen-free conditions. All procedures were approved by the Ethics Committee of the First Affiliated Hospital of Zhejiang University (approved no. 20211438), in accordance with the National Institutes of Health Guide for Care and Use of Laboratory Animals (NIH Publications, No. 8023, revised 1978).

### Chemicals and reagents

Shenfu injection was obtained from China Resources Sanjiu (Ya’an) Pharmaceutical Co. (Shenzhen, China). Lipopolysaccharide (LPS) was from Sigma-Aldrich (St. Louis, MO). Antibodies (p44/42 MAPK(Erk1/2)(3A7)mouse mAb, phospho-p44/42 MAPK(Erk1/2)(thr202/Tyr204), p38, p-p38) used for western blotting (WB) were from Cell Signalling Technology (Danvers, MA). Claudin-4 antibody was obtained from Santa Cruz Biotechnology (Santa Cruz, CA). Enzyme-linked immunosorbent assays (ELISA) kits for cytokines were purchased from Abcam (Cambridge, MA). TUNEL Apoptosis Assay Kit was obtained from Roche (Basel, Switzerland). Click-iT Plus EdU Assay Kit was from Thermo Fisher Scientific (Waltham, MA).

### MLE-12 cell culture and stimulation

*In vitro* cultured MLE-12 cells were routinely maintained with F12 and Dulbecco’s Modified Eagle Medium (DMEM) medium (1:1) containing 10% foetal bovine serum (FBS) and 1% penicillin-streptomycin in a humidified CO_2_ incubator at 37 °C with a 5% CO_2_ atmosphere. LPS treatment of MLE-12 cells was 10 µg/mL for 6 h. Shenfu was added to MLE-12 cells at 10 µl/mL.

### Transfection of the MLE-12 cell line

*Cldn4* siRNA (si-claudin-4) or *Cldn4* overexpression plasmids (OE-claudin-4) were obtained from Genepharm (Shanghai, China). MLE-12 cells were transfected with *Cldn4* (Claudin 4) siRNA or *Cldn4* overexpression plasmids using the Lipofectamine 3000 reagent according to the manufacturer’s instructions.

### Haemorrhagic shock, resuscitation, and LPS-induced acute lung injury model

Eight-week-old male C57BL/6 mice (normal or ALI model mice; *n* = 6 per group) were fasted for 12 h with free access to water. Before surgery, the mice were anaesthetized using intraperitoneal subcutaneous injection of chloral hydrate. Catheters for monitoring arterial pressure was placed into right jugular vein, right carotid artery, and left femoral artery. Jugular vein cannulation was used for resuscitation. Carotid artery cannulation was used for blood pressure monitoring. Femoral artery cannulation was used for blood withdrawal. Haemorrhagic shock was performed by blood withdrawal with the reduction of the mean arterial blood pressure (MAP) to 30 mmHg, lasting for 30 min. Then, the mice were resuscitated by transfusion of two volumes of Ringer’s Lactate over 20 min. Subsequently, the catheters were removed, blood vessels were ligated and incisions were closed. The LPS-induced ALI model was established by airway administration of LPS (500 µg/100 μL per mouse). Saline or Shenfu injection (30 mL/kg) was administered via the dorsal vein of the mouse penis in the control or treatment mice group.

Mouse lung, large intestine, and whole blood were collected at day 7 to evaluate the therapeutic effect of Shenfu injection. Pathological changes of the lung and large intestine tissues were detected using haematoxylin and eosin (H & E) staining. Tissue apoptosis was detected using a terminal deoxynulceotidyl transferase nick-end-labeling (TUNEL) assay. The dry to wet weight ratio of the lung was detected by weighing. Myeloperoxidase (MPO), intercellular adhesion molecule-1 (ICAM-1) activity, and claudin-4 protein levels in the lung and large intestine tissues were detected. Inflammatory cytokines in the bronchoalveolar lavage fluid were detected using ELISAs. Blood samples were collected and serum creatinine and uric acid were measured. Survival of the ALI mice was monitored for 7 days.

### Acute gastrointestinal injury model

Eight-week-old male C57BL/6 mice (*n* = 6 per group) were allowed free access to 3% dextran sulphate sodium (DSS) in drinking water for the 7 days to induce AGI. Then, the successfully modelled mice with diarrhoea and weight loss were further used to induce ALI via haemorrhagic shock, resuscitation, and LPS administration for the therapeutic evaluation of Shenfu injection.

### TUNEL assay

Paraffin-embedded tissue slices on slides were dewaxed, washed with phosphate buffered saline (PBS) and digested with proteinase K in a wet box for 30 min at 37 °C. After washing with PBS, the slides were dipped in TUNEL reaction mixture, and then incubated for 1 h at 37 °C. After washing, the sections were incubated with converter-POD for 30 min at 37 °C in the wet box, and then washed with PBS. Subsequently, the sections were stained with DAB chromogenic reagent for 10 min at room temperature. The slices were lightly counterstained with haematoxylin, dehydrated with ethanol. Signals were observed under a microscope.

### Myeloperoxidase (MPO) activity assay

Neutrophil infiltration was measured using an MOP assay. At the end of the experiment, the left lungs (*n* = 6) were removed from the mice of all groups and homogenized. The homogenates were then used to measure MPO activity. The MPO ELISA kit was purchased from Sigma Aldrich. Briefly, the weighed lungs were frozen and homogenized in homogenate medium. The homogenates were then centrifuged at 13,000 rpm at 4 °C for 5 min. The supernatants were added to the supplied buffer with 1% H_2_O_2_ and O-dianisidine dihydrochloride solution, according to the manufacturer’s instructions. The mixed solution was then placed in a spectrophotometer and read at 450 nm.

### EDU (5-ethynyl-2′-deoxyuridine) incorporation assay

Cell proliferation was measured using an EDU assay. EDU was diluted in dimethyl sulfoxide (DMSO) to a concentration of 10 mM. EDU was added to parallel cultures growing exponentially in 30 cm^2^ Petri dishes to a final concentration of 10 μM. Cells were then fixed with 2% formaldehyde in PBS for 2 min, permeabilized using 70% EtOH and washed with PBS-Tween20 (PBST) three times. EDU-substituted DNA was detected by an EDU Alexa Fluor 488 Imaging kit from Thermo-Fisher Scientific (Waltham, MA). Antifade mounting medium with 4′,6-diamidino-2-phenylindole (DAPI) was added to the cells and the slides were sealed for observation under a fluorescence microscopy.

### Quantitative real-time reverse-transcription polymerase chain reaction (qRT-PCR)

Total RNA was extracted from mice tissues using a TRIzol RNA extraction kit (Thermo Fisher Scientific), and reverse transcribed into complementary DNA using Prime Script RT kit (TaKaRa, Dalian, China) based on the manufacturers’ instructions. The qRT-PCR was performed using ABI 7500 Real-Time PCR System (Applied Biosystems, Foster City, CA) and Takara SYBR Premix Extaq kit (TaKaRa, Dalian, China). Primers were synthesized by Shanghai Sangon (Shanghai, China) with the following nucleotide sequences:

- claudin4/m-F, 5′-AGTCATGGTGTGCTGAGTGA-3′
- claudin4/m-R, 5′- AACCCGTCCATCCACTCTAC-3′

### Western blotting analysis

Total proteins from mouse tissues or mouse epithelial cells were extracted with Radioimmunoprecipitation assay (RIPA) buffer according to the manufacturer’s instructions. Protein concentrations were determined using a BCA protein assay kit. A total of 40 µg of protein was separated using a 10% SDS-PAGE gel and transferred to a polyvinylidene fluoride (PVDF) membrane. After blocking with 5% skimmed milk for 2 h, the membrane was incubated with primary antibodies (1:1000) overnight at 4 °C. The membrane was then washed three times with TBST and incubated with secondary antibodies (1:2000) at room temperature for 2 h. Then the expression of protein was detected by electrochemiluminescence (ECL) assay.

### Immunohistochemistiy (IHC)

The tissue samples of mice were embedded in liquid paraffin and cut into 4 μm paraffin sections. The primary antibodies were added at 4 °C overnight. Next, after incubated with corresponding secondary antibodies at 37 °C for half an hour, the sections were stained with DAB plus kit based on manufacturer’s protocol.

### ELISA

Specific concentrations of serum creatinine, uric acid, IFN-γ, IL-2, TNF-α, IL-6 and IL-1β were measured according to the manufacturer’s instructions by ELISA.

### Statistical analysis

Experiments were performed in triplicate at least three times separately. All results are presented as the mean and standard deviation (SD). An unpaired t test was used to compare the difference between two groups of data. Statistical significance was defined with a *p*-value <0.05. A log-rank (Mantel-Cox) Test was performed to analyze the survival rate. GraphPad Prism software (GraphPad software, San Diego, CA) was used to perform all the statistical analyses.

## Results

### Shenfu injection ameliorates haemorrhagic shock, resuscitation and LPS-induced ALI in mice

**Figure 1.**
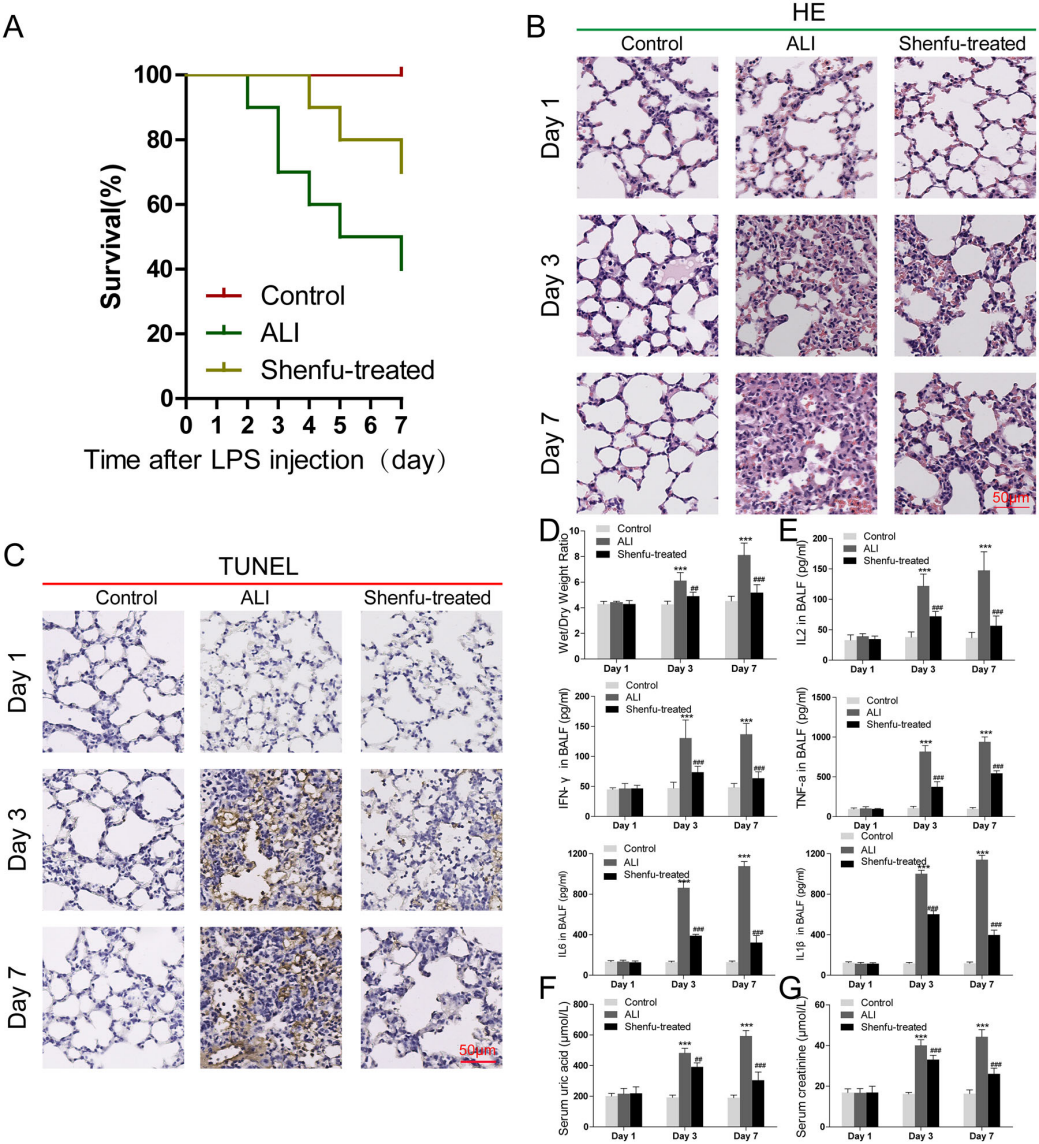
The therapeutic efficacy of Shenfu injection in mice with haemorrhagic shock, resuscitation, and LPS-induced ALI. The therapeutic efficacy of Shenfu injection in mice with ALI was investigated. A haemorrhagic shock, resuscitation, and LPS-induced ALI mouse model was built with 8-week-old male C57BL/6 mice. The control group, ALI group, and the Shenfu-treated ALI group each had 6 mice. (A) Survival of the mice as monitored for 7 days (20% body weight loss). (B) Pathological changes in tissues observed using HE staining. (C) Cell death in lung tissue observed using TUNEL staining. (D) Acute tissue injury in the lung evaluated using the wet/dry weight ratio of the lung. (E) Bronchoalveolar lavage fluid (BALF) from lung were collected from the mice. The concentrations of inflammatory cytokines IL2, IFN-γ, TNF-α, IL-6 and IL-1β were detected using ELISA. Blood samples were collected, and the concentrations of uric acid (F) and serum creatinine (G) were measured. Data are shown as means ± SD, *p < 0.05; ***p < 0.001 [determined using one-way analysis of variance (ANOVA) with Tukey comparisons].

First, we built a haemorrhagic shock, resuscitation and LPS-induced ALI mouse model to evaluate the potential therapeutic effect of Shenfu injection. In the ALI model group, only 40% of the mice survived after 7 days; however, the injection of Shenfu significantly increased the survival of ALI mice to about 70% (Figure 1(A)). To study the role of Shenfu during the development of ALI, the lungs of the ALI model mice were collected on days 3 and 7 and analyzed histologically. The HE staining result of normal mouse lung (Figure 1(B)) showed the intact structure of healthy tissue with clear alveolar cavities, and the structures of the alveolar septa were uniform and consistent. However, in the ALI mouse lung on day 3, the structural destruction was obvious. The size of the alveoli changed because of alveolar wall was edoema, and the alveolar space was collapsed with emphysema formation. Inflammatory cells infiltrated the alveolar interstitium. The severe histological tissue damage of the ALI group was enhanced on day 7. In the Shenfu injection ALI group, injury patches, vascular congestion, and inflammatory infiltrates were all significantly relieved on day 3 compared with those in the saline-injected ALI group. Further recovery was observed recovery on day 7. TUNEL staining of the lung tissues showed consistent results (Figure 1(C)). To evaluate the role of Shenfu on the alveolar leakage caused by ALI, the lung wet/dry weight ratio of the control, disease, and treated mice were measured (Figure 1(D)). In the ALI group, the lung wet/dry weight ratio increased on days 3 and 7, and Shenfu injection almost completely rescued the changes, suggesting that leakage cause by the destruction of the alveolar epithelial barrier in ALI was significantly ameliorated by Shenfu injection. Furthermore, we collected bronchoalveolar lavage fluid (BALF) from the mouse lungs and measured the concentrations of inflammatory cytokines (Figure 1(E)). The ELISA results also showed that the concentrations of IFN-γ, IL-2, TNF-α, IL-6 and IL-1β were elevated in the ALI mice. However, Shenfu injection inhibited the increased secretion of these cytokines, indicated its anti-inflammatory aspect. We concluded that Shenfu has a therapeutic function towards LPS-induced ALI via its anti-inflammation role and protective effect against tissue injury. In addition, we checked systemic damage by ALI in other organs, such as the kidney. The increase in uric acid and the associated elevation of creatinine levels in serum are generally accept as markers for the deterioration of renal function; therefore, we measured concentrations of serum uric acid and creatinine (Figure 1(F,G)). The increased uric acid and creatinine in diseased mice suggested the development of ALI-related kidney injury. However, Shenfu injection suppressed the increase of uric acid and creatinine on day 3, which was enhanced on day 7, whereas in the ALI group, renal damage was worse compared with that on day 3. The result suggested that the protective role of Shenfu to ALI has a systemic aspect.

### Shenfu injection regulated p38/ERK phosphorylation and cell viability in epithelial cells cultured *in vitro* with LPS stimulation

**Figure 2.**
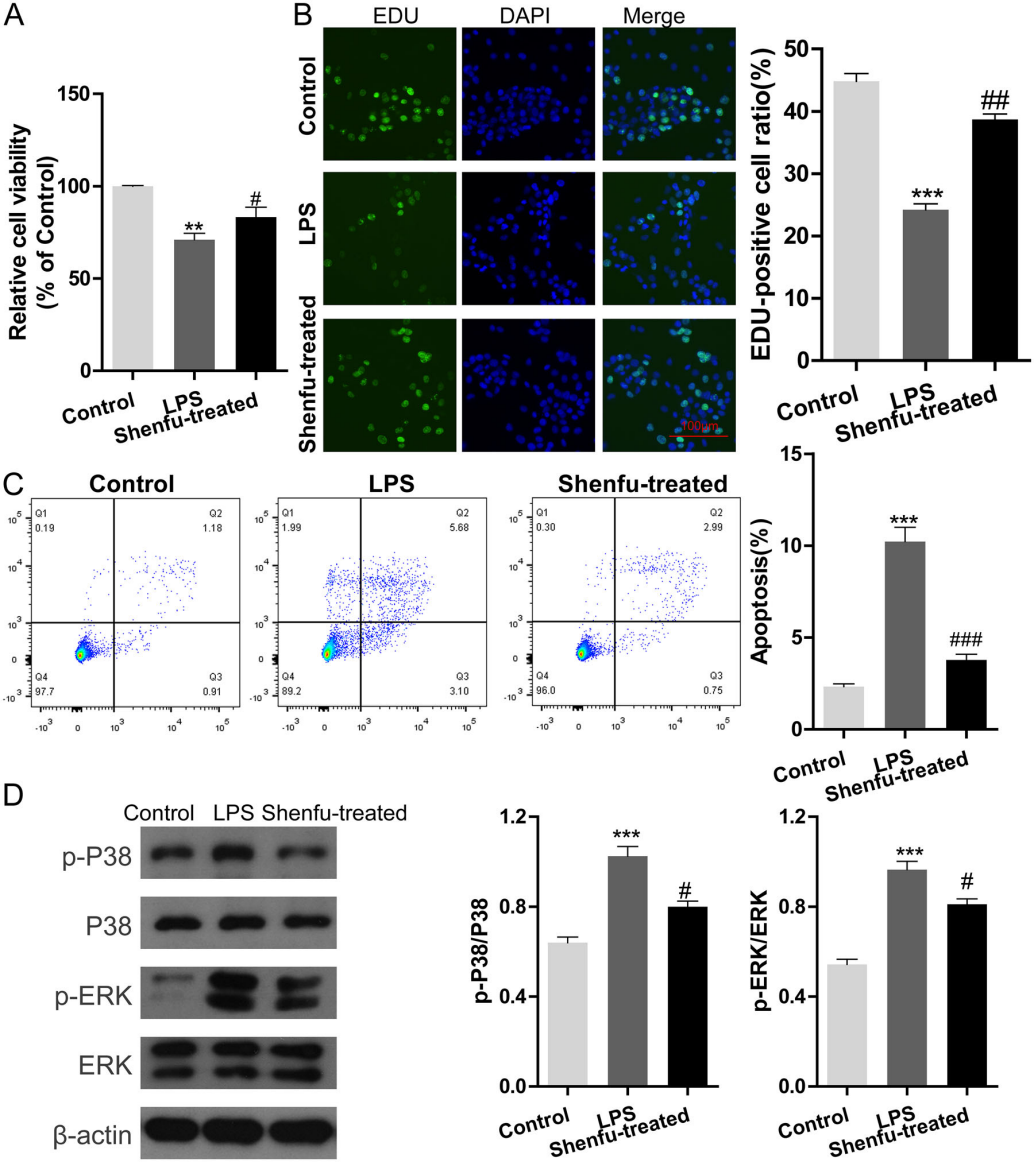
The effect of Shenfu injection on LPS stimulated MLE-12 cells. The effect of Shenfu injection on LPS-stimulated MLE-12 cells was investigated. Epithelial cell damage of ALI was modelled by LPS stimulation of MLE-12 cells *in vitro*. Cells in the ALI group were treated with 10 µg/mL LPS for 6 h Cells in the Shenfu-treated group were treated with 10 µg/mL LPS and 10 µl/mL Shenfu injection for 6 h. (A) Relative cell viability. (B) MLE-12 cell viability detected using EDU staining. DAPI was used for nuclear staining. Ratios of EDU-positive cells were analyzed. (C) Cell death was monitored by flow cytometry and the death ratios are presented. Effect of LPS stimulation and Shenfu treatment on the levels of phosphorylated ERK and p38 (D) was detected by western blot. Data are shown as means ± SD, ***p < 0.001; ^##^*p* < 0.05; ^###^*p* < 0.001 (determined by one-way ANOVA with Tukey comparisons).

To determine whether the protective effect of Shenfu acts via epithelial cells, we used the *in vitro* cultured mouse epithelial cell line MLE-12. LPS serves as a mediator for lung–gut crosstalk, and played an initiation role to induce tissue injury in the ALI model in the present study. Therefore, we treated MLE-12 cells with LPS to simulate ALI in *in vitro* cultured epithelial cells. We checked the effect of Shenfu on cell death and proliferation. CCK-8 and EDU staining were used to detect and quantify cell proliferation in MLE-12 cells. Figure 2(A) shows that while LPS stimulation of MLE-12 cells caused a 29% decrease in relative cell viability, Shenfu treatment almost eliminated this reduction. Further analysis of the results showed that the proportion of EDU positive (proliferating) MLE-12 cells decreased from 44% to 24% under LPS stimulation, whereas Shenfu treatment recovered the proportion to above 38% (Figure 2(B)). Flow cytometry was used to monitor cell death. The results showed the LPS induced 10% death of MLE-12 cells, which was reduced by Shenfu treatment to about 4% (Figure 2(C)). LPS simulation induces the activity of JNK p38 and ERK MAPKs in epithelial cells, including those in the lung and intestine (Cario et al. 2000; Jin and Jin 2018), and the MAPK pathways regulate cell death and proliferation. Therefore, we asked if Shenfu treatment could regulate MAPK activity. WB (Figure 2(D)) showed that in MLE-12 cells, the levels of phosphorylated p38 and ERK were low in the control group. LPS induced the phosphorylation of p38 and ERK, whereas Shenfu treatment suppressed it. The MAPK family plays an important role in complex cellular programs like proliferation and apoptosis (Zhang and Liu 2002), these results suggested that upregulation of cell viability by Shenfu treatment in ALI might be related to the suppression of MAPK activities.

### Shenfu injection upregulated claudin-4 expression in lung epithelial cells of ALI mice

**Figure 3.**
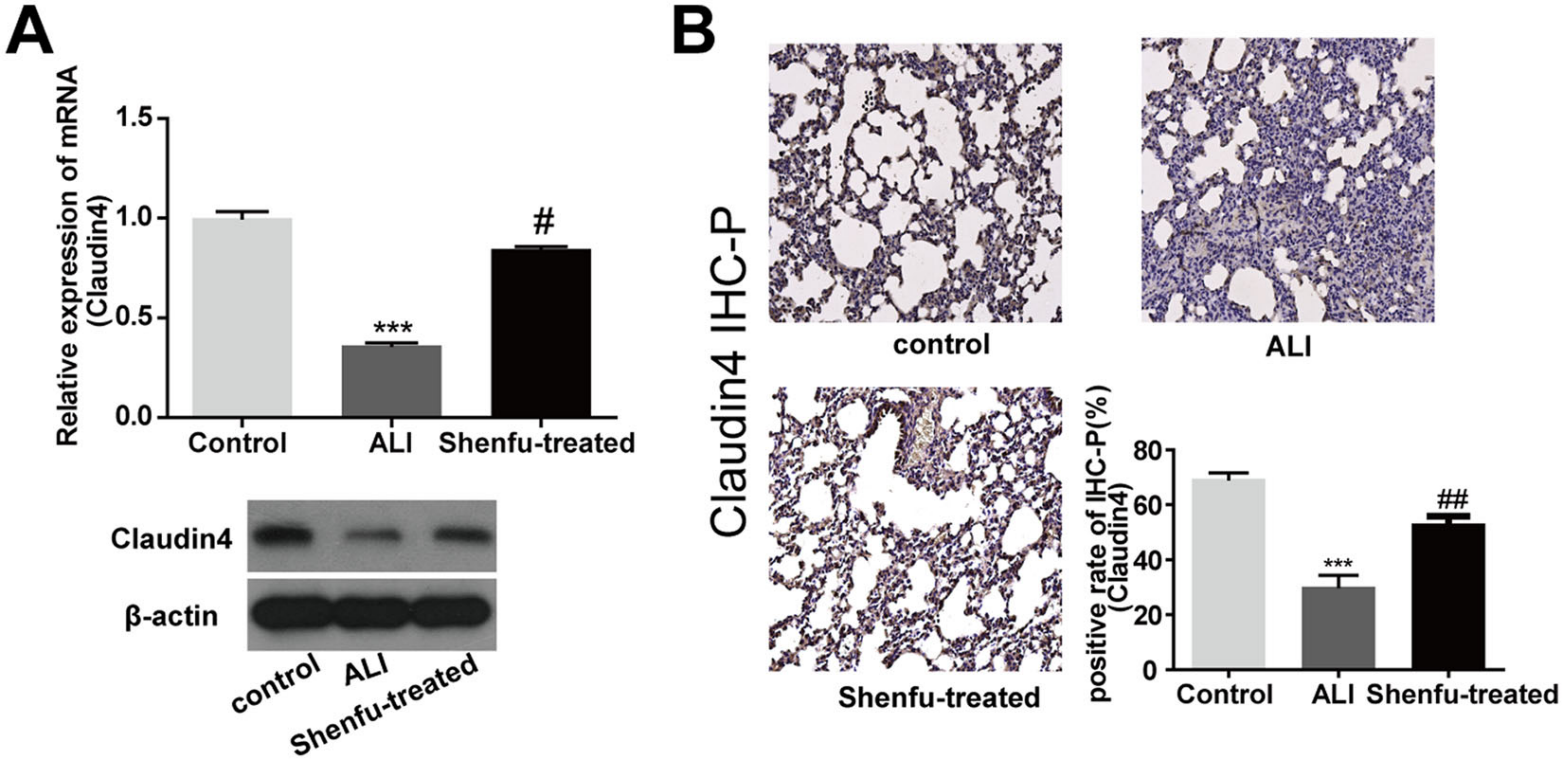
The regulatory effect of Shenfu injection on claudin-4 expression in mouse lungs with haemorrhagic shock, resuscitation, and LPS-induced ALI. The regulatory effect of Shenfu injection on claudin-4 expression in mouse lungs with ALI was investigated. The haemorrhagic shock, resuscitation, and LPS-induced ALI mouse model was built using the same protocol as in Figure 1. At day 7, lungs of mice from the three experimental groups were collected. (A) *Cldn4* mRNA transcription levels in lung tissues as determined by qRT-PCR, and claudin-4 protein levels in lung tissues as determined by WB. Pathological changes in tissue were observed using HE staining. (B) Claudin-4 expression in cells of lung tissue sections were detected using IHC-P staining and the percentages of claudin-4 positive cells are shown. Data are shown as means ± SD, ^#^*p* < 0.01; ^##^*p* < 0.05; ***p < 0.001 (determined using one-way ANOVA with Tukey comparisons).

Claudin-4 plays a central role in the maintenance of the normal epithelial barrier structure and function, as well as in recovery of the epithelium from tissue injury in ALI; therefore, we evaluated the effect of Shenfu on claudin-4 expression in lung tissue (Figure 3(A)). Along with the lung epithelial damage caused by ALI, the *Cldn4* mRNA level decreased more than 60%. However, Shenfu injection strongly rescued the loss of *Cldn4* expression. The changes in claudin-4 protein levels matched its mRNA levels in the lung, suggesting that the underlying mechanism was related to transcriptional regulation. The IHC-P staining results of claudin-4 in mouse lung tissues also revealed that ALI caused a decrease in claudin-4 levels, which was rescued by Shenfu injection, and showed claudin-4 accumulation surrounding the alveolar cavity (Figure 3(B)). These results further suggested that the Shenfu-induced increase in claudin-4 in alveolar epithelial cells contributed to the therapeutic effect on ALI.

### Manipulation of claudin-4 expression in *in vitro* cultured epithelial cells controls the effect of Shenfu treatment on cell viability and MAPK activity

**Figure 4.**
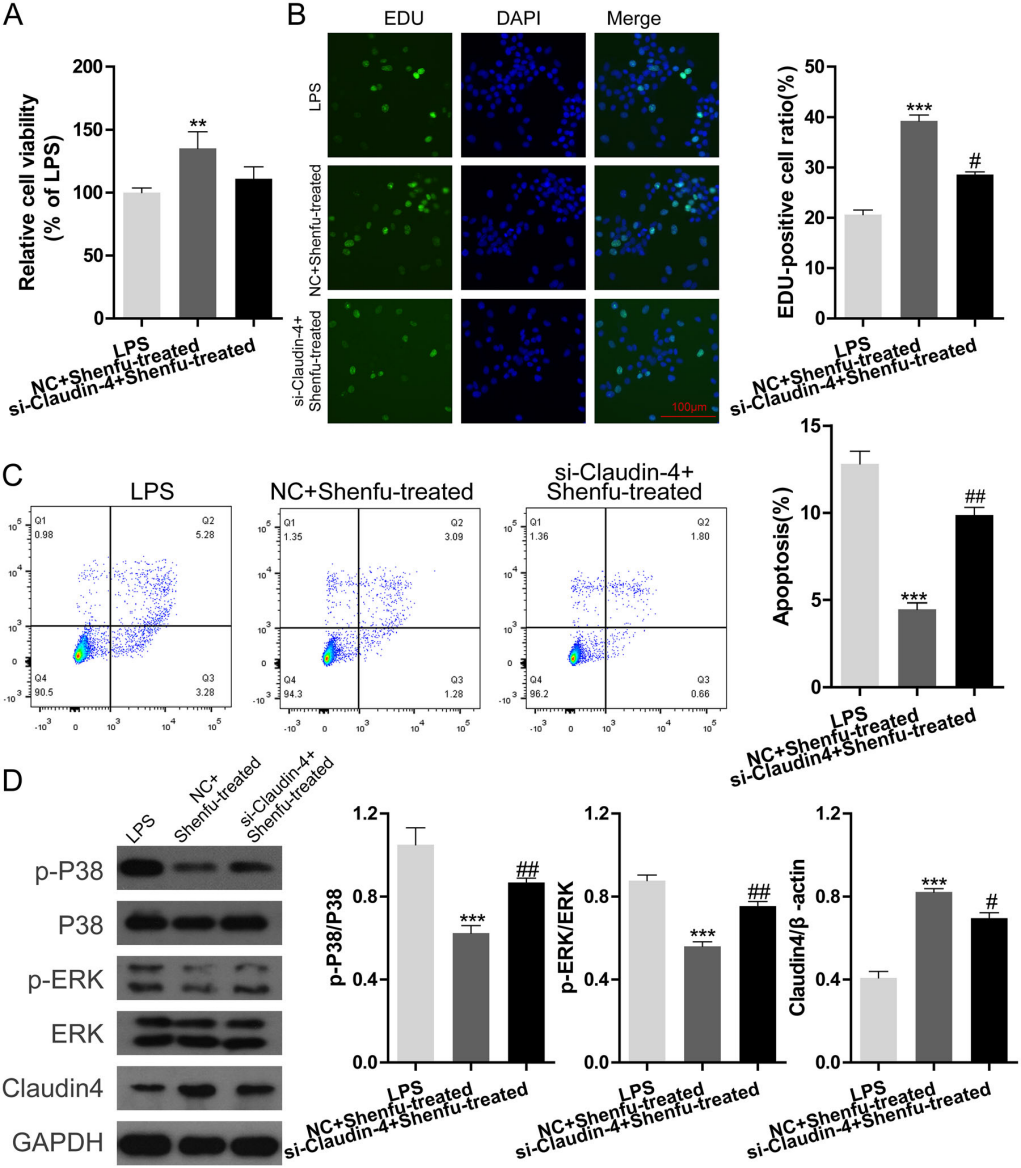
Knockdown of claudin-4 expression in epithelial cells affected the therapeutic effect of Shenfu. The effect of manipulating claudin-4 expression on the effect of Shenfu in regulating MLE-12 cell viability was investigated. Negative control or *Cldn4* siRNA was transduced into MLE-12 cells. The combined effect of manipulating claudin-4 levels and addition of Shenfu to MLE-12 cell viability under LPS stimulation was monitored by relative cell viability (A) and EDU staining (B). (C) Cell death was monitored using flow cytometry and the death ratios are shown. (D) Effect of LPS stimulation and Shenfu treatment on the level of phosphorylated ERK and p38, as detected using western blotting. Data are shown as means ± SD, ***p < 0.001 (determined by one-way ANOVA with Tukey comparisons).

**Figure 5.**
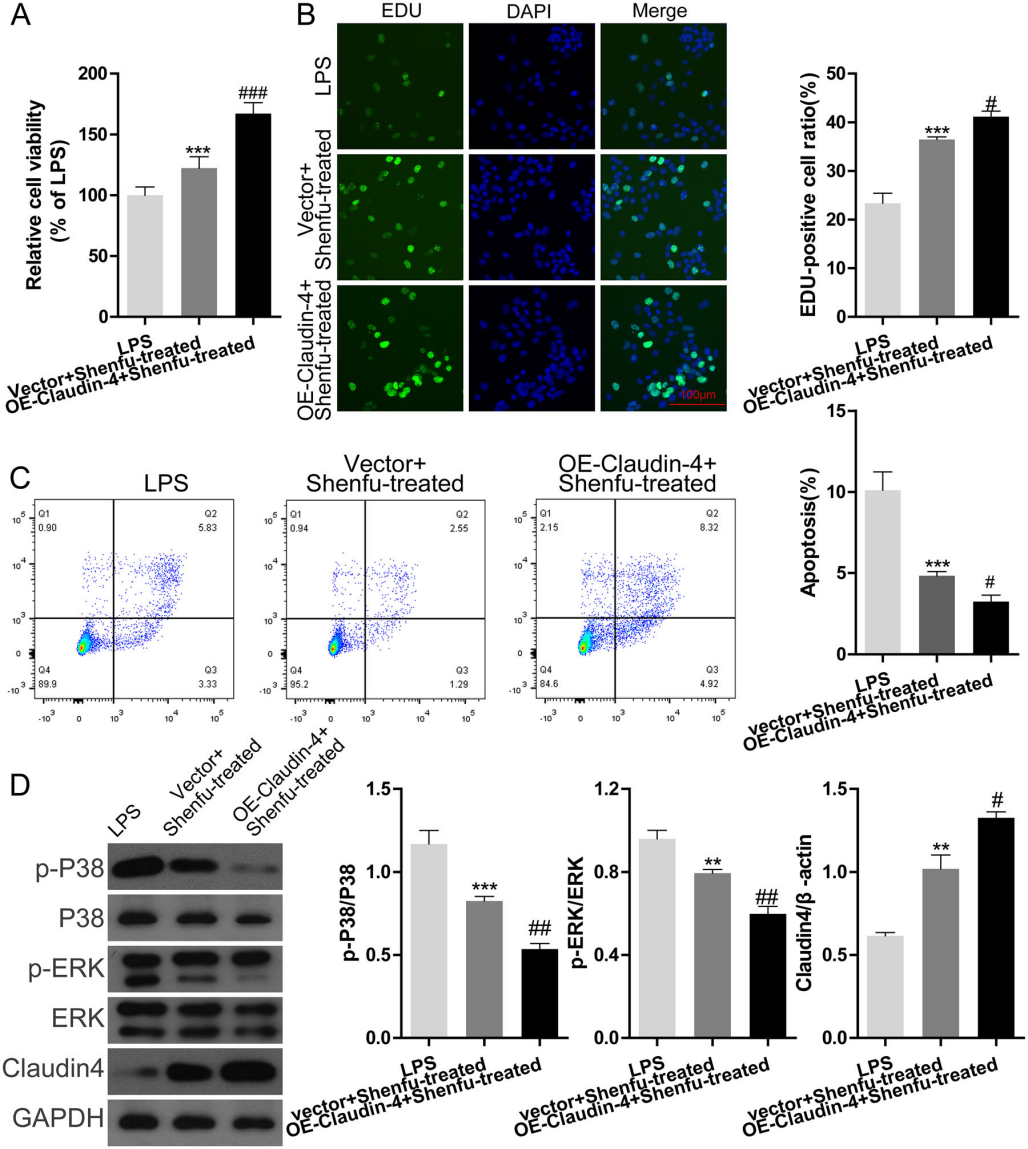
Overexpression of claudin-4 in epithelial cells affected the therapeutic effect of Shenfu. The effect of manipulating claudin-4 expression to alter the effect of Shenfu in regulating MLE-12 cell viability was investigated. Vector or *Cldn4* overexpression plasmid was transduced into MLE-12 cells. The combined effect of manipulating claudin-4 expression and addition of Shenfu to MLE-12 cell viability under LPS stimulation was monitored by the relative cell viability (A) and EDU staining (B). (C) Cell death was monitored using flow cytometry and the death ratios are shown. (D) Effect of LPS stimulation and Shenfu treatment on the level of phosphorylated ERK and p38, as detected using western blotting. Data are shown as means ± SD, ***p < 0.001 (determined by one-way ANOVA with Tukey comparisons).

Claudin affects viability and function of epithelial cells and our data showed that Shenfu treatment increased MLE-12 cell viability. Therefore, we wondered if claudin-4 was required in this process. We assessed the effect of Shenfu in MLE-12 cells with different levels of claudin-4 expression via *Cldn4* siRNA or *Cldn4* overexpression plasmid. CCK-8 and EDU staining showed that Shenfu treatment-induced rescue of the suppressed cell viability under LPS stimulation was diminished by silencing of *Cldn4* (Figure 4(A,B)). By contrast, *Cldn4* overexpression in MLE-12 cells enhanced the function of Shenfu in the rescue of cell viability (Figure 5(A,B)). Results for LPS-induced MLE-12 cell death were consistent with the EDU staining results. *Cldn4* silencing counteracted the effect of Shenfu to suppress LPS-induced cell death (Figure 4(C)), whereas *Cldn4* overexpression enhanced the suppressive function of Shenfu (Figure 5(C)). However, surprisingly, the WB results showed that manipulation of *Cldn4* expression changed phosphorylation states of p38 and ERK (Figure 4(D)). LPS-induced activation (phosphorylation) of p38 and ERK could not be suppressed by Shenfu in *Cldn4*-silenced cells, whereas overexpression of *Cldn4* enhanced the suppressive effect of Shenfu on p38 and ERK phosphorylation (Figure 5(D)). These implied activities of MAPKs were regulated by Shenfu in a claudin 4-dependent manner, further suggesting the existence of a feedback loop between MAPK activation and claudin expression.

### Shenfu diminished the intestinal tissue damage complicated with lung injury of the ALI mice

**Figure 6.**
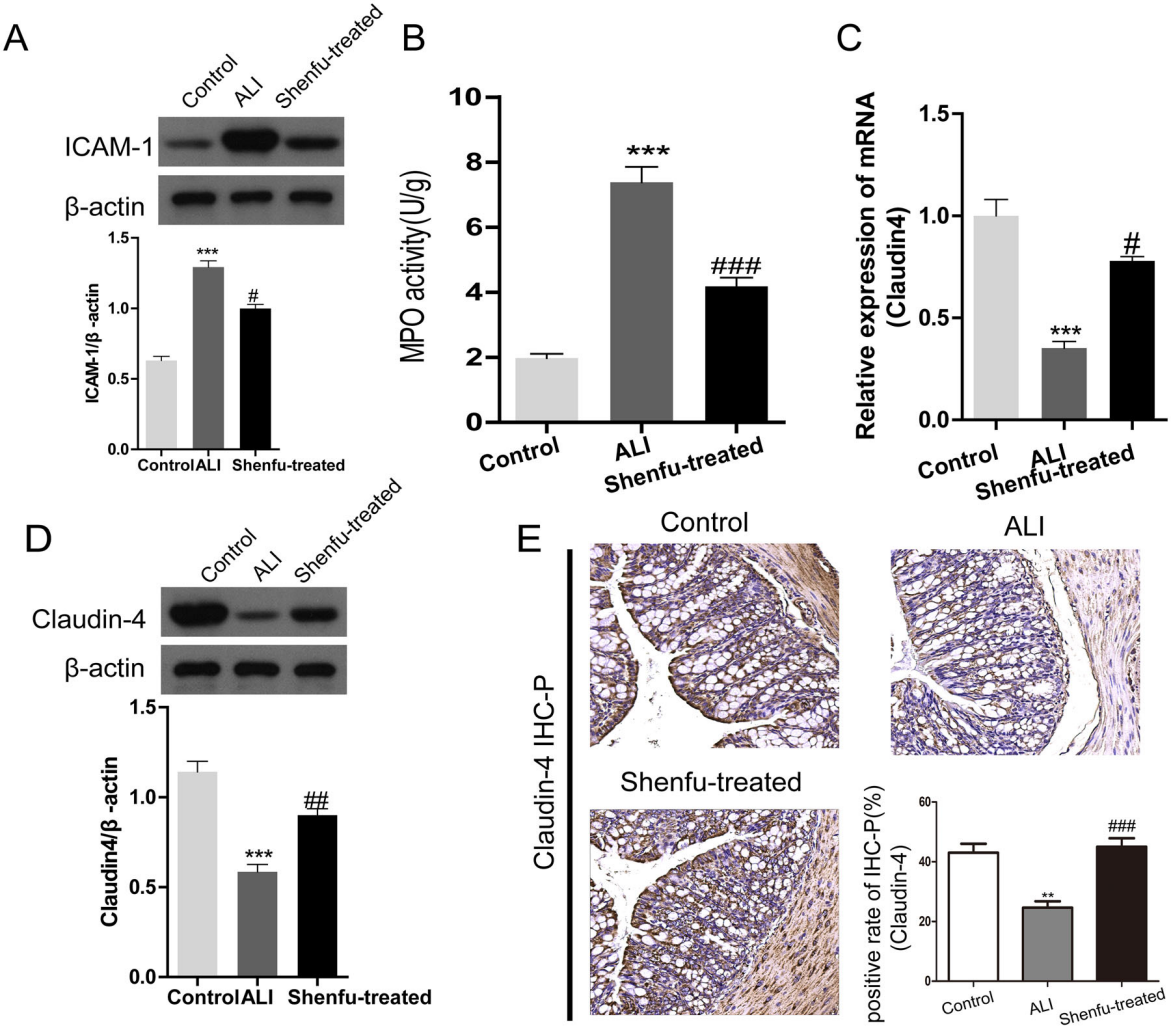
The therapeutic efficacy of Shenfu injection on tissue damage and claudin-4 levels in the large intestine in mice with ALI. The therapeutic efficacy of Shenfu injection on large intestine damage caused by ALI was investigated. The haemorrhagic shock, resuscitation, and LPS-induced ALI mouse model was the same as in previous experiments. The large intestines of ALI mice were collected on day 7. (A) ICAM-1 protein levels as detected using WB. (B) MPO activities in the large intestines of ALI mice. (C and D) mRNA or protein levels of claudin-4 in intestinal tissue as detected using qRT-PCR or WB. (E) Pathological changes and claudin-4 expression in the large intestine tissue were evaluated using IHC-P staining. Data are shown as means ± SD, **p < 0.01; ***p < 0.001; ^###^*p* < 0.001 (determined using one-way ANOVA with Tukey comparisons).

Next, we assessed the pathological state of the gastrointestinal tissue of the ALI model mice. ICAM-1 is a cell surface glycoprotein and an adhesion receptor known to play the central role in inflammation (Bui et al. 2020). WB of large intestine tissue showed that ICAM-I was upregulated in the ALI mice (Figure 6(A)). MPO activity is a very sensitive marker that reflects both neutrophil accumulation and neutrophil activation. It is regarded as one of the most sensitive markers of tissue inflammation available. MPO activity in the colonic mucosa was also analyzed for the biochemical evaluation of tissue damage. Consistent with the ICAM-1 expression results, MOP activity was elevated in the large intestine of the ALI mice, suggesting ALI was accompanied by gastrointestinal injury in these mice (Figure 6(B)). However, in the Shenfu-treated ALI mice, intestinal MOP activity and ICAM-1 expression were suppressed, implying diminished tissue inflammation and injury after Shenfu injection (Figure 6(A,B)).

We also checked the claudin-4 mRNA and protein expression levels in the large intestine of the mice. Accordingly, a 50% decrease in claudin-4 expression was detected in the intestinal tissue of the ALI mice and a 55%-increase in claudin-4 expression was induced by Shenfu injection (Figure 6(C,D)). The result was further confirmed by IHC-P staining of claudin-4 in tissue sections of large intestine (Figure 6(E)). In the control group, claudin-4 expression in the intestinal epithelium was high, with 40% positively stained cells. In the ALI group, claudin-4 expression was reduced to 20% positively stained cells, whereas Shenfu injection rescued the expression of claudin-4 in the intestinal epithelium to 40%. These results indicated that Shenfu might have systemic protective effect to multiple organs in mice with ALI.

### Shenfu diminished the tissue damage of ALI mice exacerbated by induced AGI

**Figure 7.**
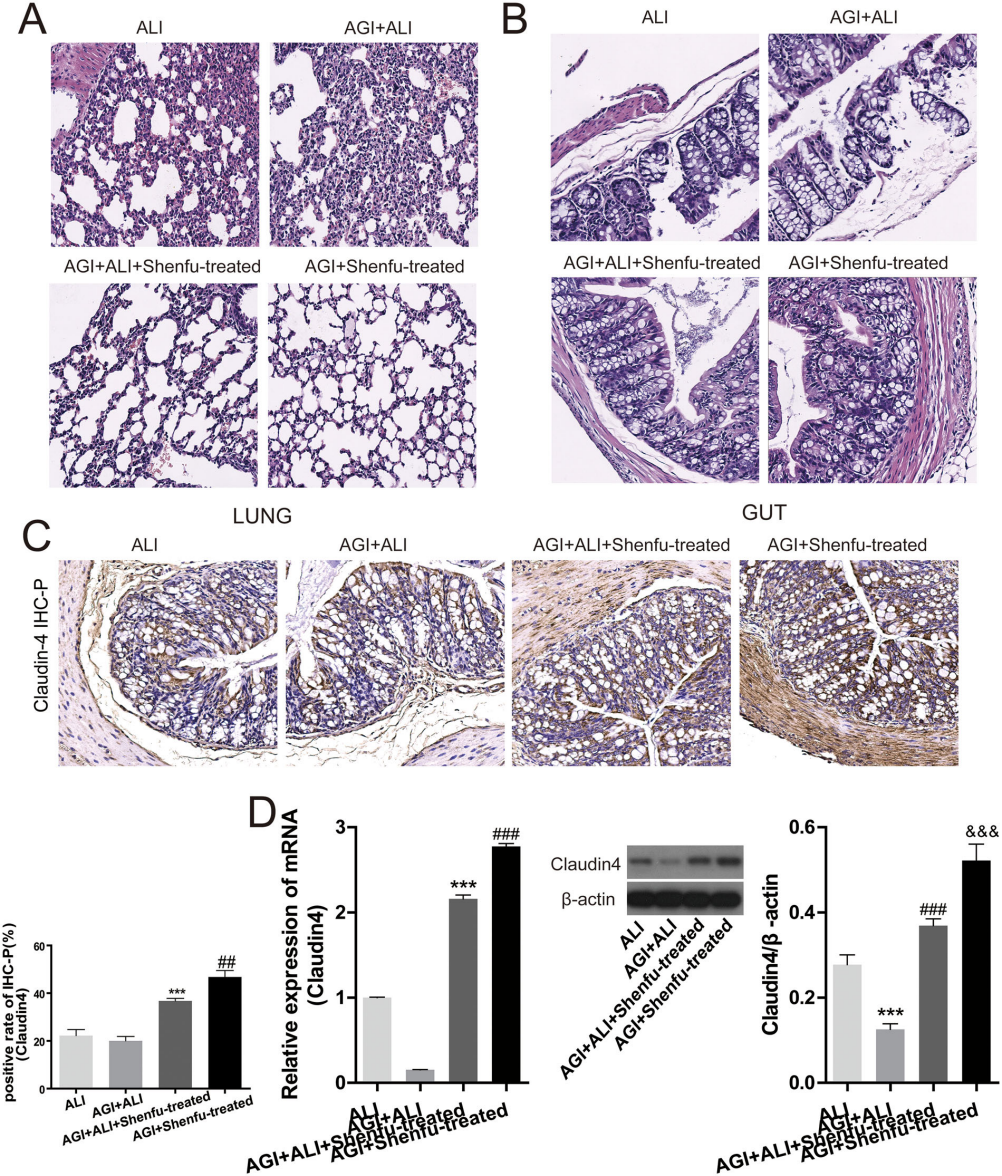
The therapeutic effect of Shenfu injection on lung and intestinal tissue damage of mice with ALI exacerbated by AGI. The therapeutic efficacy of Shenfu injection in mice with ALI exacerbated by AGI was investigated. Mice were divided into four groups. For the ALI group, the disease model was the same as in the previous experiments. For the AGI or AGI + ALI mouse groups, AGI mice were induced using 3% DSS added in drinking water for 7 days. The mice in the AGI group received Shenfu injection. For the AGI + ALI group, the successfully modelled AGI mice were further used to induce ALI with haemorrhagic shock, resuscitation, and LPS administration. Then AGI + ALI mice were divided into two groups for control or Shenfu injection. 7 days after ALI induction and Shenfu injection, the lungs and large intestine of the mice were collected. The pathological condition of the lungs (A) or intestine (B) as evaluated using HE staining. Claudin-4 protein levels in the large intestine as detected using IHC-P staining (C), the positive expression rates are shown. (D) mRNA or protein levels of claudin-4 in intestinal tissue as detected by qRT-PCR or WB (D). Data are shown as means ± SD, **p < 0.01; ***p < 0.001; ^###^*p* < 0.001 (determined using one-way ANOVA with Tukey comparisons).

A previous study reported that intestinal epithelial injury may also aggravate ALI (Zhou and Liao 2021). Therefore, we asked if the protective role of Shenfu in the intestines also contributed to the therapeutic effect against ALI. We compared the lung injury between the ALI mouse model and the AGI + ALI mouse model to test our hypothesis. The HE staining results showed that the severe lung tissue damage caused by ALI was indeed exacerbated by AGI in the AGI + ALI group compared with that in the ALI group (Figure 7(A)). However, in the AGI only and AGI + ALI groups treated with Shenfu, with or without induced ALI, sections of lung tissues showed almost normal morphology (Figure 7(A)). Corresponding to the pathological state in the lung, HE staining of the large intestines also showed that the intestinal damage in mice caused by AGI was exacerbated by ALI. Shenfu injection significantly prevented this ALI-associated intestinal injury (Figure 7(B)). IHC-P staining results (Figure 7(C)) also showed claudin-4 expression was almost completely suppressed by the combined effect of AGI and ALI. However, treatment with Shenfu in either the AGI or AGI + ALI mice rescued the loss of claudin-4 expression in the intestines. The result was further confirmed by WB and mRNA-qRT-PCR (Figure 7(D)). These results suggested that Shenfu could protect against both lung and intestinal epithelial damage in AGI-exacerbated ALI.

## Discussion

In past decades, extensive research into the pathophysiology of patients with ALI revealed many of the underlying cellular and molecular mechanisms of the causes and development of the disease. Based on this understanding, many new therapeutic strategies were developed against ALI. However, none of them could significantly decrease the mobility and mortality of patients with ALI. Chinese traditional medicine is a practical and theoretical approach to diseases developed over thousands of years. Although there is a lack of rigorously designed studies and experiments, Chinese traditional medicine doctors still have accumulated knowledge about disease pathology and herbal pharmacology within their medical experiences. Shenfu is a Chinese herb generally used against medical problems including stroke, heart disease, and respiratory diseases. Its therapeutic effect and its mechanism have been studied. Based on the anti-inflammatory and tissue protective aspects of Shenfu, we proposed that it could have the potential to treat ALI. Patients usually develop ALI via the combined consequences of intrapulmonary injury or infection and extrapulmonary factors, such as systemic severe infection, trauma, and shock. In the present study, we built a haemorrhagic shock, resuscitation, and LPS-induced mouse ALI model. In this model, intravenous administration of LPS was the intrapulmonary initiation factor, and haemorrhagic shock and resuscitation served as the extrapulmonary stimulation factors. Theoretically, it should be a better model to simulate ALI with multiple causes than the ALI mouse models induced by single factors. Our ALI mouse model provided a more comprehensive basis to evaluate the therapeutic effect of Shenfu. The results of our study showed that Shenfu injection significantly diminished the tissue damage in the lungs of the ALI mice. Regarding the extrapulmonary tissue damage that accompanies ALI, such as in the kidney and intestine, our study also suggested that Shenfu injection has a systemic protective effect. Moreover, we induced AGI in the ALI mice. The results showed that AGI exacerbated the symptoms in the lungs and large intestines of the ALI mice. Shenfu injection of the AGI + ALI mice significantly reduced the lung and intestinal epithelium damage, suggesting that the partial therapeutic effect of Shenfu in lung tissue was related to its protective effect on the intestinal epithelium.

Claudin family proteins are the major structural components of the tight junction of all epithelia and endothelia (Gunzel and Yu 2013). They constitute both paracellular barriers and pores, and determine the permeability properties of epithelial and endothelial cells. Claudin-4 is predominantly expressed in mouse and human lung tissue (Kaarteenaho-Wiik and Soini 2009). In the lung, claudin-4 is important to maintain the barrier function, preventing the leakage of electrolytes and fluid into the alveolar space (Kaarteenaho-Wiik and Soini 2009; Wray et al. 2009). Our results demonstrated that the therapeutic effect of Shenfu in ALI acts via the upregulation of claudin-4 expression in epithelial cells. Claudin4 expression was upregulated by Shenfu at both the mRNA and protein levels, suggesting that the mechanism is related to transcriptional regulation. Furthermore, our data suggested that p38 and ERK activities were regulated by Shenfu in a claudin 4-dependent manner. LPS simulation induced the activity MAPKs in epithelial cells, including those in the lungs and intestines (Yeh et al. 2014; Xing et al. 2019; Zhu et al. 2020), and MAPKs also provide inhibitory signals that regulate function of the epithelial barrier and the expression of claudins in mammary epithelial cells (Carrozzino et al. 2009). Furthermore, a study showed that claudin-4 expression in the lungs of ALI model mice was regulated by the PKC and JNK MAPK pathway (Wray et al. 2009), which implied that LPS stimulation of epithelial cells might suppress claudin-4 expression in MAPK-dependent manner. Taken together with our result that MAPK activity was regulated by claudin-4 expression, these conclusions suggested a feedback loop of MAPK activity and claudin-4 expression that regulates epithelium function.

The gene encoding claudin-4 is conserved in human, chimpanzee, dog, cow, mouse, chicken, and even zebrafish, suggesting the universality and conservation of the claudin 4-related mechanism in the maintenance of the epithelial barrier function and the recovery of epithelial damage. Considering the suggested conserved role of claudin-4 across species, we have reason to believe that the therapeutic effect of Shenfu in mice and humans should be the same. Consequently, Shenfu has a great potential as an effective drug for patients with ALI. However, unlike western medicine, which has a single component and clear effect, Chinese herbal medicine and compound medicine have complex network characteristics of multiple components and pathways. At present, most of the research on Chinese herbal medicine is based on network pharmacology, which reveals the relationship between drugs, genes and diseases by analyzing the network regulation effect and PPI network (Tang et al. 2022; Tian et al. 2022). Based on the conclusions of our study, we will carry out further research on the effective ingredients of Shenfu in the future. We believe that further studies will promote the drug discovery and the development of therapeutics for ALI.

## Conclusions

This study demonstrated that Shenfu ameliorated LPS-induced ALI *in vivo* and *in vitro*. Mechanistically, Shenfu mediated the protective effect by upregulating claudin-4 expression. In addition, Shenfu could protect against both lung and intestinal epithelial damage in acute gastrointestinal injury-exacerbated ALI. The results revealed the therapeutic effect and the underlying mechanism of Shenfu injection in ALI, indicating its clinical potential to treat patients with ALI.

## Data Availability

All data generated or analyzed during this study are included in this published article.
